# Value delivery in green consumption: the effect of advertisement value proposition on consumer perception and purchase intention

**DOI:** 10.3389/fpsyg.2024.1339197

**Published:** 2024-01-23

**Authors:** Yan Zhang, Jiaqi Liu, Xiaoyong Li

**Affiliations:** School of Economics and Management, Beijing Forestry University, Beijing, China

**Keywords:** value co-creation, green advertising, temporal framing, advertisement believability, S-D logic

## Abstract

Amid the expanding green consumer market, companies are increasingly emphasizing their eco-friendly contributions in advertising. This study delves into the value delivery process within green consumption, guided by the service-dominant logic (S-D logic). A survey-based experiment with 589 responses reveals that the information frame in advertising significantly impacts consumer value perceptions and purchase intentions. Notably, the temporal frame of advertising plays a pivotal role, influencing consumers' value perceptions. Future framing enhances emotional value perceptions, while past framing promotes environmental value perceptions. This research enhances our understanding of value delivery mechanisms in green consumption and holds valuable implications for marketers of sustainable products.

## 1 Introduction

An increasing number of environmental tribunals, industry associations, and environmental groups are actively engaged in formulating and disseminating environmental regulations. In response, enterprises proactively disclose their pro-environmental achievements through avenues such as green advertisements and environment, social, and governance (ESG) reports (Zheng M. H. et al., 2023). With the proliferation of green information in marketing communication, investigation into how consumers comprehend enterprises' green claims has emerged as a significant research topic (Bi et al., 2023). Existing research suggests that advertisements' appeals (functional vs. emotional), framing (gain vs. loss), and visual effects (such as color and image proximity) may influence consumers to either choose or resist green products (Sheng et al., 2021; Su and Li, 2022; Wenting et al., 2022; Zhang et al., 2023). Enterprises increasingly require precise advertising settings to alleviate consumer skepticism and promote their recognition of green products.

Numerous theories and psychological mechanisms play a pivotal role in shaping consumers' interpretation of green advertising. These include self-construal theory, self-identity theory, self-efficacy theory, and self-control theory (Zheng et al., 2022; Ogiemwonyi and Jan, 2023a; Sung and Lee, 2023; Zheng C. et al., 2023; Zheng M. H. et al., 2023). Following exposure to advertisements, consumers form a preliminary identity and attitude toward products, influencing their decision on whether to engage in consumption (Li et al., 2022; Wang, 2022; Ogiemwonyi and Jan, 2023b).

Studies consistently indicate that consumers generally respond positively to green advertisements, though occasional skepticism arises from concerns about greenwashing (Kwon et al., 2023; Muralidharan et al., 2023). The variation in perceptions of identical advertising content offers an opportunity to apply the S-D logic, which views advertising as a communication channel for conveying firms' value propositions (Verleye and Reber, 2022). Consumers assess these propositions and form their distinctive value perceptions. The extent of the gap between firms' value propositions and consumers' value perceptions ultimately determines the effectiveness of green marketing (Edvardsson et al., 2011).

Studies following the S-D logic predominantly center on the impact of green value co-creation, such as green product innovation involving stakeholders, on firm performance (Diaz-Perdomo et al., 2021; Tian et al., 2023). Limited research investigating how consumers are influenced by enterprise propositions and subsequently develop a willingness to participate in green value co-creation (Font et al., 2021). This offers a gap to further explore the value delivery process in green marketing.

The first step in value delivery involves elucidating the information framework of the value proposition. This study considers two dimensions of proposition framework. The first dimension is believability, which plays a crucial role in addressing the central concern of green advertising: alleviating consumers' skepticism and fostering trust (Osburg et al., 2019). The second dimension is temporal frame, which serves to distinguish the impact of consumer engagement degree on value perception under the S-D logic. Previous research suggests that specific message frames, such as distant vs. proximal temporal frames, could effectively engage consumers with environmental sustainability initiatives (Salnikova et al., 2022). This study introduces a past vs. future temporal frame that differentiates value propositions associated with production process from those associated with consumption process in the product life cycle (Kim et al., 2021). Value claims that materialize prior to purchase, such as cleaner production certifications and sustainable material labels, are presented in a past-framed manner, wherein consumers cannot intervene (Iovino et al., 2023). Conversely, value claims that call for consumer engagement in reuse, recycling, or adopting low-energy modes during product usage are framed in relation to the future.

Then, consumers' value perceptions play a mediating role in translating value propositions into value co-creation intentions. This study distinguishes emotional value perceptions from environmental value perceptions, aiming to explore whether dimensions of value propositions exert diverse impacts on consumers' value perceptions. Finally, this study considers purchase intentions as a form of value co-creation intentions, acknowledging that purchase intentions hold the potential to deliver economic value for firms, value-in-use for consumers, and environmental benefits for society.

This study aims to attain two main objectives: (1) to formulate a framework delineating the value delivery process in green marketing; (2) to enhance insights into the influence of temporal framing on consumers' perceptions and purchase intentions. Theoretically, the research findings extend the applicability of S-D logic to the realm of green advertising. Moreover, research suggests that the temporal frame does not directly impact purchase intention but rather exerts an indirect influence by shaping consumers' value perceptions. This highlights the role of value perception in fostering green value delivery. Practically, the research suggests that past framing can be employed to enhance consumers' environmental value perception, while future framing can be utilized to enhance their emotional value perception. Enterprises could strategically use appropriate temporal frames with credible messages to enhance the persuasiveness of green communication.

## 2 Conceptual framework and hypotheses development

### 2.1 Theoretical framework

The S-D logic, a well-established concept in marketing literature, posits that value is co-created through the interaction between providers and consumers in service exchanges, with its determination lying in the hands of consumers (Skalen et al., 2015). Apathy toward products or services may arise due to a lack of resonance with consumer values (Font et al., 2021). To address this challenge, enterprises must craft effective value propositions and employ communication channels such as social media, advertising, and service encounters to convey their propositions to customers (Sung and Lee, 2023).

Considering value co-creation as the overarching goal, the S-D logic delineates a strategic process for the creation and delivery of value. Firstly, firms strategically determine the information to share and what value they promise for their offerings in their propositions (Verleye and Reber, 2022). Then, through service encounters or advertisements, firms communicate these value propositions to consumers (Jäger and Weber, 2020). During the evaluation of value propositions, consumers generate the concept of value-in-use, where product value is subjectively determined and may exceed the scope of the value proposition (Ranjan and Read, 2016). The alignment between value propositions and perceptions is imperative for achieving mutual gains in transactions (Edvardsson et al., 2011), prompting firms to carefully model consumers' perceptions and find a compromise between customer desires and provider offerings (Rintamaki and Kirves, 2017). Lastly, the intention for value co-creation, encompassing actions such as product purchase, brand engagement, knowledge sharing, or brand advocacy, is engendered when consumers affirm the value of service, signifying that the value gained surpasses the associated costs (Luo et al., 2022).

### 2.2 Value delivery in green consumption

Green products are defined as products that have a limited negative impact on the environment throughout their life cycle, encompassing stages such as research and development, production processes, use, and post-use disposal. Consequently, green consumption denotes a psychological inclination among consumers to opt for and purchase environmentally friendly products as an alternative to conventional ones. In the context of green consumption, green purchasing is regarded as a typical value co-creation behavior, because it can create value for enterprises, society, and consumers themselves (Nadeem et al., 2021). Applying the S-D logic, factors that lead to green purchase intention include both firms' value propositions and consumers' value perceptions. High-quality propositions, whether in the form of persuasive evidence or storytelling communications, enhance consumers' understanding of product value, simultaneously mitigating their risk aversion and skepticism, thereby bolstering their confidence in purchase decision-making (Gasparin et al., 2022; Hamzah et al., 2022). Meanwhile, the green perceived value serves as an effective indicator of green purchase intention (Zheng et al., 2022; Zheng C. et al., 2023; Zheng M. H. et al., 2023). An innovative aspect of this study involves illustrating the value delivery process in green consumption within the context of the S-D logic. A theoretical innovation of this study lies in demonstrating the value transfer process in green consumption following the S-D logic. This framework serves as a valuable tool for marketing practitioners, enabling them to discern the impact of crucial variables in a firm's value proposition on consumer perception and purchase intention.

### 2.3 Advertisement believability and purchase intention

As grown wary of greenwashing, many consumers are suspicious about companies' environmental claims. Believability has become a key research topic in green advertising (White et al., 2019; Kim et al., 2022). As a dimension of information quality, believability is defined as the extent to which a viewer perceives information presented in the message as truthful and credible (Lee et al., 2002). Firms usually add environmental certifications and eco-labels in advertisements to enhance persuasion and induce consumer adoption (Cian et al., 2020; Lima et al., 2023; Rathee and Milfeld, 2023). Advertisement believability plays a key role in shaping customers' positive brand evaluation and purchase decisions (da Luz et al., 2020; Ogiemwonyi, 2022; Rahman and Bang, 2022). Research also suggests the effects of advertisement variables (such as message style or appeals) on green purchases are completely mediated by advertisement believability (Jäger and Weber, 2020). Therefore, we propose the following hypothesis:

H1: Advertisement believability positively affects consumers' green purchase intention.

### 2.4 Value perception as mediators

The S-D logic suggests that products' value is proposed by firms but determined by consumers (Laudien et al., 2023). Green value perception is a general evaluation of a product's environmental friendliness which plays a primary role in shaping consumers' buying behavior (Ahmad and Zhang, 2020). In green consumption research field, the widely recognized green perceived value dimensions include biosphere value, egoistic value, altruistic value, and hedonic values (Steg et al., 2014). By combining two theory stream, Koller et al. (2011) suggest that ecological value is the core of green consumption which lead to value perceptions including social value, economic value, emotional value, and functional value dimensions. Different combinations of value dimensions show some similarities. From the perspective of value creation, the environmental value and social value of products reflect altruistic tendencies, while the functional, emotional, and hedonic values serve self-appraisal needs (Ogiemwonyi et al., 2020; Suphasomboon and Vassanadumrongdee, 2022). In this study, we consider two aspects of green value: environmental value and emotional value. The emotional value concerns about pleasure and enjoyment of consumers the environmental value concerns about reduction of pollution or resource waste in the biosphere (Li et al., 2021; Suphasomboon and Vassanadumrongdee, 2022).

The theory of information overload suggests that only the information that attracts consumers' attention and trust can affect their value evaluation (Hu et al., 2022). Perceived value plays a mediating role between consumer information processing and decision-making (Uzir et al., 2021). When facing an unfamiliar green product, consumer skepticism about product function effectiveness or greenwash can lead to negative value perception (Hou and Sarigollu, 2022). Thus, believable value propositions make consumers confident in the making right evaluations and purchase decisions (Kim et al., 2022). A higher believability may increase the perceived value to consumers (Martinelli and De Canio, 2021). Therefore, we propose the following hypothesis:

H2a: Advertisement believability positively affects consumers' environmental value perceptions.
H2b: Advertisement believability positively affects consumers' emotional value perceptions.

Green value perception and the resulting green attitude jointly affect consumers' green purchase intention and revisit intention (Lavuri, 2022; Riva et al., 2022). Specifically, different values may have different effects on consumer attitude and behavior (Muralidharan et al., 2023). The belief that choosing a green product will help in reducing pollution, conserving resources, and improving the environment has a positive association with green purchasing (Dhir et al., 2021; Upadhyay and Kamble, 2023). Emphasizing the moral significance of green products will make consumers value their moral sense and encourage them to be good and environmentally responsible people, therefore driving them to purchase green products (Cheung and To, 2019). When consumers recognize the value-in use, high green values stimulate them to accept green offerings (Laudien et al., 2023). Therefore, we propose the following hypothesis:

H3a: Consumers' environmental value perceptions positively affect their purchase intention.
H3b: Consumers' emotional value perceptions positively affect their purchase intention.

### 2.5 The temporal framing effect

Temporal framing is a sort of information framing that affects the perceptions of information recipients by different temporal focus (past, present, and future) and distance (near and distant) (Baird et al., 2021). This study takes the product delivery time as the time node which separates past and future temporal direction (Patala et al., 2016; Kim et al., 2021). A future frame makes commitments on goals that have not been implemented while a past frame focuses on claiming achievements the firm already made (Tuan et al., 2023). In green propositions, past-framed messages reveal certain environmental benefits a product owns such as the number of pollution and carbon emissions reduction during production. Future-framed messages focus on the long-term environmental benefits in the post-purchase stage (Zhang, 2022) such as the benefits of product usage or the recycling process.

Different temporal orientations may lead to various value perceptions among consumers. In some circumstances, advertisements with past temporal framing evoke a nostalgic feeling (Barnwell et al., 2022). People show more trust and need less evidence to identify the proposed value in past temporal messages compared to future ones (Zhang, 2022). Typical past-framed messages such as environmental labels and certifications with confirmed environmental benefits are found to be positively influencing environmental value perceptions (Iovino et al., 2023). Stakeholders with high uncertainty avoidance prefer messages with real examples of firms' achievements, thereby past-framed information would be convincing (Tuan et al., 2023). From an information certainty perspective, claims about prognostic benefits may be exaggerated (Kwon et al., 2023). Therefore, we propose the following hypothesis:

H4a: Past temporal framing positively moderates the influence of advertisement believability on environmental value perceptions.

Aspirations about an ideal and unlimited possibilities represents future orientation of personnel (Zhang, 2022). Future-framed messages echo this orientation thus could evoke people's positive emotions such as hopeful and excitement (Robinson and Veresiu, 2021; Zhang, 2022). On the one hand, future-oriented messages attract consumers' positive evaluations, thereby promoting their identification of their future self (Silvi and Rosa, 2021). On the other hand, future-oriented messages often require consumers to participate in the green value-creation process which enhances their value contribution perception (Winterich et al., 2019). The ritual vitality resulting from consumer co-creation may generate additional desired outcomes and emotions (Bradford and Sherry, 2023). In terms of influencing consumer cognition, future-framed information is more emotionally appealing than past-framed information (Kim et al., 2021). Therefore, we propose the following hypothesis:

H4b: Future temporal framing positively moderates the influence of advertisement believability on emotional value perceptions.

**Figure 1:**
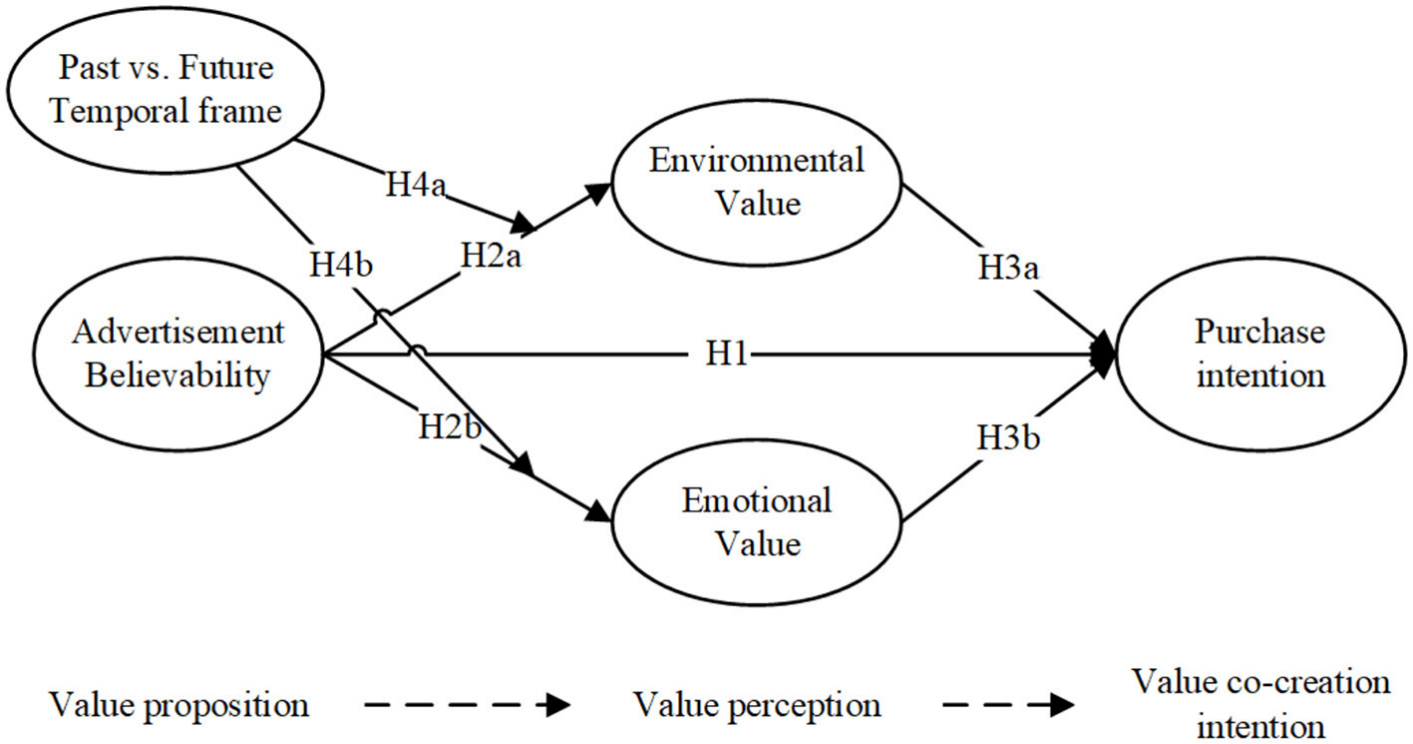
The proposed research model.

In summary, following the value-co-creation framework, the hypotheses proposed are shown in Figure 1.

## 3 Methodology

### 3.1 Experimental design

With the development of green marketing practices, consumers' green purchase intention on apparel, food, hospitality, and transportation product or services has been widely studied. Given that this study concerns consumers' perception and behavior tendency on value propositions, food consumption is chosen as the research background because it is familiar to consumers. Respondents can more easily understand the preset purchase need and decide whether to purchase relying on the information provided in food consumption scenarios than other less common consumption scenarios.

In designing experimental stimulus (see Table A1), we write a brief introduction on a fictitious plant-based oat milk. Oat milk is well-known for its low-carbon and environmentally friendly properties thereby consumers consider its green value more than the flavor or nutritive value in consumption. Compared with organic foods, the vegan food is a more subdivided food category that is more relevant to the green value that this study focuses on are more compatible with the environmentally friendly attributes and the perceived value classification concerned in this study.

In reference to advertisements of well-known oat milk brands, we classify value propositions with different temporal frames into two groups. One group shows the green value achieved in product production (past framing), such as carbon reduction and resource conservation in the production process. The other group shows green value could achieve during product consumption (future framing), such as recyclable packages and energy-saving levels during usage.

### 3.2 Measurement and research methods

A randomized survey-based experiment is used to test the hypotheses. The survey contains four parts. First, participants are randomly assigned to one stimulus conditions (past or future framing) before answering questions. Second, participants need to answer two questions for manipulation test and the 7-point Likert scale (1 = strongly disagree; 7 = strongly agree) is adopted for rating each question. One is: “This advertisement claims the product's green attribute in the production stage and focuses on the past-achieved green benefits.” The other is: “This advertisement claims the product's green attribute in the consumption stage and focuses on future commitments on green benefits”.

Third, all measurements of key constructs are adapted and developed from previous studies and (7-point Likert scale, see Table A2). Advertisement believability is measured by four items developed from Kumar et al. (2021) and Li et al. (2021). Environmental value is measured by four items developed from Dhir et al. (2021) and Hu et al. (2022). Emotional value is measured by four items adapted from Roh et al. (2022). Green purchase intention is measured by four items developed from Li et al. (2021) and Hu et al. (2022). When adapting the measurements, similar questions in both scales were removed, and the wording and structure of the questions were modified and standardized to align with the stimuli materials created for this study.

Fourth, five factors that may influence green purchase intention are selected as control variables. Participants are asked to offer some demographic information (gender, age, level of education, income) and evaluate the degree of green knowledge they know (7-point Likert scale).

This study employs covariance-based structural equation modeling (CB-SEM) to assess the direct effects between variables, due to the model's strong theoretical foundation and our capability in getting the required sample size. Additionally, the PROCESS macro is utilized to examine the multiple mediation effects, as it offers a matching model set (Model 7 and Model 8) for testing and comparing mediation effects, helping identify any potential missing correlation hypotheses in the proposed framework.

### 3.3 Pretest

A pretest is conducted to check if the experimental stimulus works properly and whether measurement scales are valid. A total of 100 college students majoring in business administration were recruited for the study. Participants randomly receive one advertisement treatment. The results of one-way ANOVA confirm that participants could distinguish between the past and future framing in advertisements. Evaluation on past focus ad (*M*_past_ = 6.1, *M*_future_ = 3.54, *F* = 24.81, *p* < 0.001) and future focus ad (*M*_past_ = 3.94, *M*_future_ = 5.4, *F* = 64.68, *p* < 0.001) shows significant difference, indicating participants can understand the created advertisement treatments correctly. Meanwhile, the results of reliability and validity analysis meet the threshold standard, thereby all questions are reserved in the data collection step.

A further examination of the research framework is that we check the correlation between the temporal frame and advertising believability. As a part of the information frame, if the temporal framing is found significantly related to believability in value propositions, the research result may be distorted due to multicollinearity. By comparing the believability score generated by the factor extraction method between two groups of participants (*M*_past_ = 5.2, *M*_future_ = 5.17, *p* = 0.77), we find no significant correlations between variables. Therefore, we do not modify the hypotheses and proposed research model.

## 4 Empirical analysis

### 4.1 Data collection and description

The experiment is administered online by a commercial survey platform Credamo (www.credamo.com). The research group pays the platform 5 RMB for each respondent. The survey service provider offered 589 valid and complete responses for analysis. The statistics of respondents' demographics of are shown in Table 1. The samples basically covered groups of different ages, education levels and incomes which supports the universality of conclusions.

**Table 1:** Descriptive statistics of demographics.

**Variable**	**Value**	**Past frame (*****N*** = **295)**		**Future frame (*****N*** = **294)**	
		**Frequency**	**Percent (%)**	**Frequency**	**Percent (%)**
Gender	Male	149	50.5	125	42.5
	Female	146	49.5	169	57.5
Age	Under 20	23	7.8	22	7.5
	20–29	110	37.3	98	33.3
	30–39	128	43.4	138	46.9
	Over 40	34	11.5	36	12.2
Education level	High school or less	124	42.0	119	40.5
	Bachelor's degree	158	53.6	158	53.7
	Master's degree or above	13	4.4	17	5.8
Monthly income	5,000 RMB or less	137	46.4	146	49.7
	5,001–10,000 RMB	105	35.6	101	34.4
	10,001–15,000 RMB	42	14.2	37	12.6
	15,001 RMB or above	11	3.7	10	3.4

The manipulation validity is checked again before data processing. Participants' respond to past framing ad (*M*_past_ = 5.3, *M*_future_ = 3.48, *F* = 210.52, *p* < 0.001) and future framing ad (*M*_past_ = 3.18, *M*_future_ = 5.74, *F* = 203.92, *p* < 0.001) shows significant difference, indicating that they understand the temporal focus of provided ads when answering questions.

### 4.2 Measurement reliability and validity

The SPSS 26.0 and Amos 26.0 are adopted to test the reliability and validity of the measurements. Results are shown in Table 2. The reliability of measurements is evaluated by Cronbach's α and composite reliability (CR). Both values exceed the threshold of 0.7 (Hair et al., 2014). The convergent validity of measurements is evaluated by factor loadings (>0.7) and average variance extracted (AVE, >0.5).

**Table 2:** Reliability and validity analysis.

**Constructs**	**Items**	**Factor loading**	**Cronbach's α**	**CR**	**AVE**
Advertisement believability (AB)	AB1	0.836	0.919	0.909	0.715
	AB2	0.832			
	AB3	0.852			
	AB4	0.861			
Environmental value (EnV)	EnV1	0.787	0.884	0.901	0.694
		EnV2				0.871
		EnV3				0.818
		EnV4				0.854
Emotional value (EmV)	EmV1	0.765	0.788	0.839	0.565
		EmV2				0.729
		EmV3				0.755
		EmV4				0.758
Purchase intention (PI)	PI1	0.832	0.878	0.893	0.676
		PI2				0.840
		PI3				0.810
		PI4				0.807

The discriminant validity of measurements is evaluated following the Fornell-Larcker criterion (Fornell and Larcker, 1981) that the correlation coefficient of two constructs is less than the square root of AVE (see Table 3). In conclusion, the reliability and validity of measurement is confirmed.

**Table 3:** Discriminant validity analysis.

	**AB**	**EnV**	**EmV**	**PI**
AB	**0.846**			
EnV	0.376	**0.833**		
EmV	0.388	0.450	**0.752**	
PI	0.573	0.242	0.345	**0.822**

The diagonal bold number is the square root of AVE.

The Harmon's single-factor test is conducted to check if their exits potential common method bias (Podsakoff et al., 2003). The four factors explained 72.48% of the total variance while the first factor explained 37.28%, indicating that common method bias is not a concern in this study.

The model fit indices of past frame samples as follows: chi-square/df = 1.278; GFI = 0.95; AGFI = 0.933; RMSEA = 0.031, NFI = 0.957, IFI = 0.99, CFI = 0.99. The model fit indices of future frame samples as follows: chi-square/df = 1.429; GFI = 0.95; AGFI = 0.931; RMSEA = 0.036, NFI = 0.968, IFI = 0.99, CFI = 0.99. Overall, the model fit is satisfactory (Hair et al., 2014).

### 4.3 Hypothesis test

Path coefficients of the structural model based on data from two temporal framing groups are calculated with AMOS26.0 (results see Table 4). The effect of the control variable is not supported and therefore not reported in the table. In both models, the positive effect of believability on purchase intention (H1), believability on value perception (H2a and H2b), and value perception on purchase intention (H3a and H3b) are supported. Although respondents received different advertising stimuli, their decision-making process showed consistency. Firm-generated value propositions could be internalized as consumers' value perceptions on products. Both value propositions and value perceptions can be used to predict consumers' purchasing tendencies. To test the robustness of the results, we combine data from two temporal framing groups and do the structural equation model analysis using the joint sample. The result is similar to results shown in Table 4 in that all five hypotheses get supported.

**Table 4:** Structural equation model analysis.

**Paths**	**Past frame**		**Future frame**	
	**Std. coeff**	***P*** **-value**	**Std. coeff**	***P*** **-value**
H1: AB → PI	0.180	0.034^**^	0.192	0.000^***^
H2a: AB → EnV	0.492	0.000^***^	0.370	0.009^***^
H2b: AB → EmV	0.456	0.005^***^	0.672	0.001^***^
H3a: EnV → PI	0.391	0.001^***^	0.294	0.000^***^
H3b: EmV → PI	0.169	0.037^**^	0.170	0.003^***^

^**^p < 0.05; ^***^p < 0.01.

To verify the mediation effect, the bootstrapping method with 5,000 samples and 95% confidence interval is adopted (Preacher and Hayes, 2008). The results are shown in Table 5. Value perceptions partially mediate the relationship between firms' value proposition and consumers' co-creation intention, indicating that advertisement believability could either influence purchasing intention directly or lead to purchase intention through consumers' value confirmation. Meanwhile, both environmental value and emotional value show significant mediating effect in the value delivery process.

**Table 5:** Mediation effect analysis.

**Paths**	**Past frame**			**Future frame**		
	**Effect**	**Bootstrap CI**	***P*** **-value**	**Effect**	**Bootstrap CI**	***P*** **-value**
AB → EnV → PI	0.185	(0.105, 0.298)	0.000^***^	0.105	(0.061, 0.168)	0.001^***^
AB → EmV → PI	0.074	(0.001, 0.166)	0.047^**^	0.123	(0.061, 0.209)	0.001^***^
Indirect effect	0.259	(0.153, 0.406)	0.000^***^	0.228	(0.153, 0.330)	0.001^***^
Total effect	0.431	(0.286, 0.592)	0.001^***^	0.421	(0.311, 0.554)	0.001^***^

^**^p < 0.05; ^***^p < 0.01.

Moreover, the difference in paths' effect suggests a probable moderation effect of the temporal frame. Since the moderating variable considered in this study (past temporal frame vs. future temporal frame) is a categorical variable, we try the Chow Test using the multiple group analysis function in AMOS 26.0 to compare the coefficients of impact paths between two models (Sharma et al., 1981). The result (see Table 6) shows significant differences in coefficients with the path AB → EnV (*p* = 0.023) and AB → EmV (*p* = 0.000) between two models, indicating that temporal stimulus brings some structural changes to the model.

**Table 6:** Differences in path coefficients.

**Paths**	**Difference (future-past)**	**Two-tailed *p* value**
H1: AB → PI	0.012	0.495
H2a: AB → EnV	−0.122	0.023^**^
H2b: AB → EmV	0.216	0.000^***^
H3a: EnV → PI	−0.097	0.294
H3b: EmV → PI	0.001	0.170

^**^p < 0.05; ^***^p < 0.01.

To further verify H4a and H4b, we run moderated mediation regression analyses (Model 7, PROCESS V3.5) with SPSS 26.0. The results are shown in Table 7 (effect of control variables is omitted for insignificant). Message framing and believability show positive impact on consumer value perceptions in both models. The moderation effect of temporal framing on different value perception paths varies. Specifically, when respondents read a future-framed advertisement other than the past-framed one, the temporal focus weakens the positive impact of advertisement believability on environmental value perceptions (supporting H4a) but strengthens the positive impact of advertisement believability on emotional value perceptions (supporting H4b). The moderated mediation regression also offers evidence for the mediation effect of environmental value and emotional value variables, the direct and indirect jointly explain 59.5% of variance in green purchase intention (*R*^2^ = 0.595, *p* = 0.000). We try Model 8 to explore the probable moderating effect of temporal frame on the relationship between advertisement believability and purchase intention. The result did not show a significant impact of the interaction variable (coeff = −0.084, *p* = 0.254).

**Table 7:** Moderation effect analysis.

**Outcome variable**	**Predict variable**	***R* ^2^**	***F***	**Coeff**	**CI**	***p*-value**
EnV	AB	0.36	28.83^***^	0.62	(0.39, 0.85)	0.000^***^
	Temporal framing			0.93	(0.16, 1.70)	0.018^**^
	AB × temporal framing			−0.19	(−0.34, −0.05)	0.009^**^
EmV	AB	0.58	100.56^***^	0.48	(0.42, 0.55)	0.000^***^
	Temporal framing			0.76	(0.60, 0.93)	0.000^***^
	AB × temporal framing			0.19	(0.06, 0.32)	0.005^***^

^**^p < 0.05; ^***^p < 0.01.

**Figure 2:**
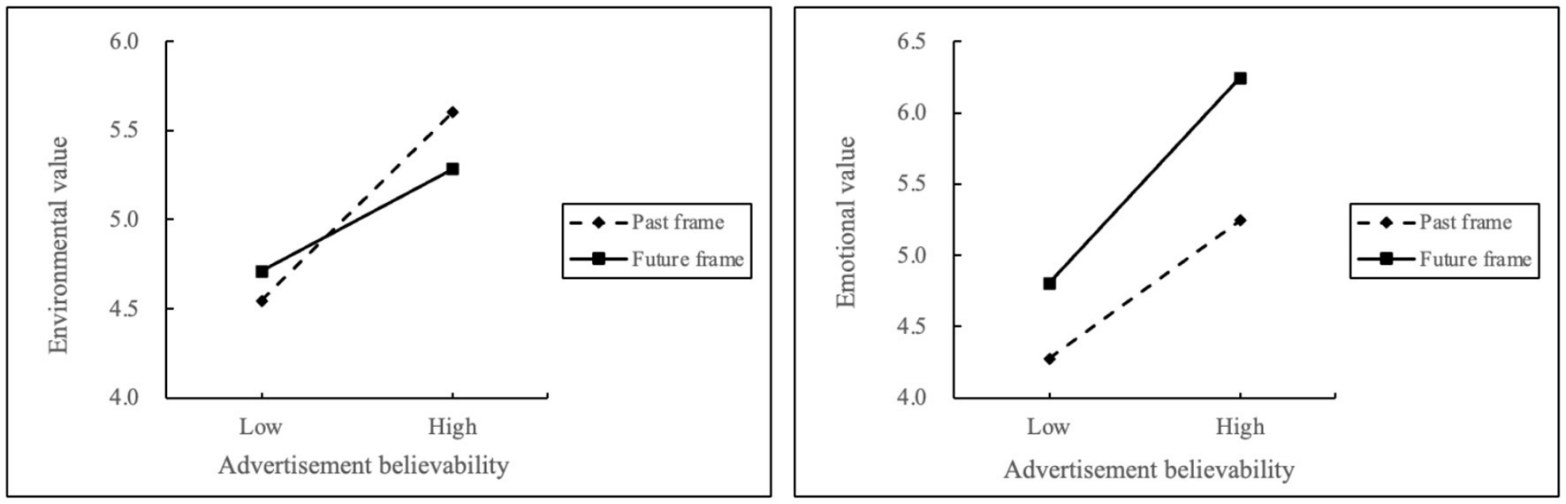
Moderation effect of temporal framing.

The visualization of interaction effect between AB and temporal framing on value perception is shown in Figure 2. Regarding environmental value as the dependent variable, the slope of regression line is milder with future temporal frame than past temporal frame. Regarding emotional value as the dependent variable, the slope of regression line is steeper with future temporal frame than past temporal frame.

In conclusion, the seven supposed hypotheses are all get supported by the survey-based experiment. Based on the S-D logic, the research framework shows how firms' value propositions influence consumers' value co-creation intention in the green consumption context.

## 5 Discussion and implications

### 5.1 Discussion

In accordance with the S-D logic, this research contributes to an enhanced understanding of the mechanisms of value delivery within the sphere of green consumption practices in China.

In terms of enterprise value proposition, prior research has underscored the impact of various informational factors, encompassing information quality (e.g., abstract vs. concrete messaging) and information framing (e.g., gain or loss framing), on consumer value perceptions (Rathee and Milfeld, 2023). Building on this, the study delves into the interaction between believability (an indicator of information quality) and temporal frame (an indicator of information framing). The findings affirm the viewpoint presented by Rathee and Milfeld (2023). Moreover, the comparison of Model 7 and Model 8 in model verification using PROCESS reveals that the temporal framing of value propositions indirectly influences purchase intention by shaping consumers' value perceptions. In contrast, the believability of value propositions directly impacts purchase intention. While previous research suggests that certain information frames, such as gain or loss frames, can influence information believability (Mulcahy et al., 2024) this study concludes, upon examining the correlations between believability and temporal frame variables, that temporal frame has no significant impact on information believability.

In terms of consumer value perception, the study highlights distinct effects of different time frames on environmental and emotional value perceptions. While existing studies on temporal framing typically adopt an classification of distant vs. proximal frame (Bradford and Sherry, 2023), this study proposes a classification based on past and future frames referring to the S-D logic. The green value proposition in the past temporal frame is initiated by enterprises, while the green value proposition in the future temporal frame necessitates consumer participation. When the realization of value does not require consumer participation, they are less likely to be aware of their personal value (Bradford and Sherry, 2023). The results affirm that consumers perceive higher environmental value with past framing and higher emotional value with future framing, validating the effectiveness of the strategy of advocating consumer participation to satisfy their emotional value in green marketing.

### 5.2 Theoretical implications

This research contributes to the application of value co-creation theory in the context of green consumption. The findings make theoretical contributions in several important aspects.

Firstly, the study highlights the impact of different temporal frames in conveying diverse value perceptions to consumers. Referring to the S-D logic, this study introduces a novel temporal frame perspective: future framing encourages consumers to pay attention to their involvement in value creation, whereas past framing directs consumers to acknowledge firms' contributions in value creation. The results illustrate that highlighting the future frame in the value proposition encourages consumers to choose products, as it may enhance their self-moral evaluation. Consequently, in the context of green consumption, consumers' awareness and preference for products can be enhanced through their participation in co-creation.

Secondly, the proposed value delivery framework establishes paths to reach a green value consensus between firms and consumers. Advertising plays a crucial role as a communication medium for firms to convey their value propositions to consumers. The quality and framework of advertisement collectively shape consumers' personalized value perceptions, ultimately influencing their purchase intentions. Consumers' purchases then contribute to the realization of value propositions and perceptions. Therefore, value is co-created to benefit consumers, firms, and the environment. Meanwhile, the framework considers consumers' perceptions of environmental and emotional value. In addition to gaining recognition for environmental value through certifications and green labels, an effective strategy to drive purchases is promoting consumer engagement in environmentally friendly behavior. This approach enhances willingness to make a purchase by increasing consumers' emotional evaluation.

### 5.3 Managerial implications

The results of this study offer important implications for marketers of sustainable products.

Firstly, firms should strategically consider consumers' value perceptions and craft tailored tailor precise value propositions in their advertisements to entice consumers. Since many green product transformations are impelled by updates in trade rules or industry standards, firms often treat green attributes as peripheral information in their advertising efforts. The research underscores the importance for firms to proactively communicate their green values in product promotions to resonate effectively with consumers. By segmenting users, firms can present different value propositions to distinct consumer groups through personalized recommendation systems. Moreover, enterprises should structure advertising information using high-credibility materials, such as well-acknowledged certificates and credible commitments, as these elements can directly amplify consumers' purchase intentions.

Secondly, incorporating narratives that highlight consumers' active participation in advertising significantly enhances their favorability. Studies indicate that consumer engagement with advertisements, such as participation in interactions or sharing advertising content, positively influences purchase intentions and brand preference. The research findings recommend an increase in advertising initiatives aimed at engaging consumers in environmentally friendly behaviors, such as recycling. Firms developing propositions that encourage consumer participation in green value creation can enhance sales by addressing consumers' emotional needs.

In summary, marketers of sustainable products should consider consumers' value perceptions, design targeted value propositions, and highlight consumers' involvement in advertising. By aligning their messaging with consumer values, utilizing personalized recommendations, and promoting consumer engagement, firms can effectively attract and engage environmentally conscious consumers, driving purchase intentions and fostering brand loyalty.

## 6 Conclusions

In conclusion, grounded in the S-D logit, this research introduces a value delivery mechanism within the context of green consumption, establishing connections between firms' value propositions, consumers' value perceptions, and purchase intentions. It highlights the crucial role of value propositions that believable propositions have a significant impact on consumer perceptions of environmental value (altruistic value) and emotional value (egoistic value). Notably, this impact is influenced by the temporal frame in which the value propositions are presented. The future frame enhances consumers' emotional value perception, whereas the past frame enhances their environmental value perception. When consumers hold high value perceptions, they are more likely to develop a green purchase intention. Meanwhile, it is imperative to acknowledge several limitations in this research. The design of stimuli may constrain the generalizability of findings to other green product categories. Additionally, the research scenarios were confined to exploring the effects of past and future time frames separately. Future studies could delve into the value delivery process when both past and future time frames are presented across diverse product categories.

## Data availability statement

The raw data supporting the conclusions of this article will be made available by the authors, without undue reservation.

## Ethics statement

The studies involving human participants were reviewed and approved by the School of Economics and Management, Beijing Forestry University. The participants provided informed consent to participate.

## Author contributions

YZ: Conceptualization, Writing – original draft. JL: Data curation, Writing – original draft. XL: Supervision, Writing – review & editing.

## Appendix

**Table A1:** Temporal framing stimulus materials.

**Past framing**	**Future framing**
Our company aims to drive a food system shift by restoring carbon, improving biodiversity, and boosting farmers' income. We believe that providing oat milk as alternatives to cow's dairy is a great way to support more sustainable diets. This oat milk product is a sustainable beverage. Each 200 ml cup of oat milk produced can reduce about 0.4 kg of carbon emissions, reduce about 1 square meter of land occupation, and save about 110 liters of water compared to the production of cow milk. A sustainable production system support oat milk in saving production resources and reducing environmental impacts.	Our company aims to drive a food system shift by restoring carbon, improving biodiversity, and boosting farmers' income. We believe that providing oat milk as alternatives to cow's dairy is a great way to support more sustainable diets. This oat milk product is a sustainable beverage. The whole packaging is recyclable. Participate in our bottle recycling program in supermarkets and convenience store. Drop the empty bottle of oat milk into the recycling bins. One ton of recycled packaging can produce 0.7 tons of recycled paper. Contribute your power to build a sustainable world.
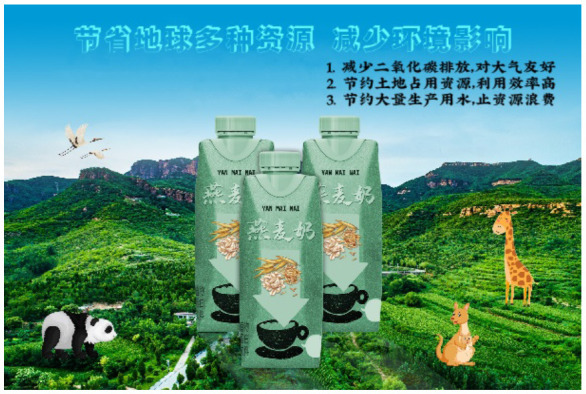
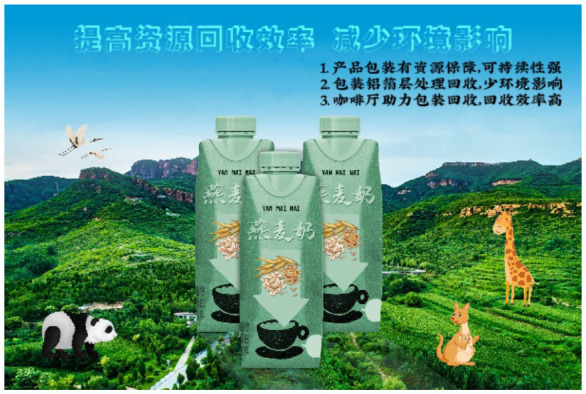

**Table A2:** Measurements.

**Constructs**	**Measurement items**	**References**
Advertisement believability	The message in the beverage advertising is honest. The message in the beverage advertising is believable. The message in the beverage advertising is reliable. The message in the beverage advertising is trustworthy.	Lee et al., 2002; Kumar et al., 2021
Environmental value	Choosing this beverage helps protect the environment. Choosing this beverage contribute to the environment. Choosing this beverage helps decrease pollution. Choosing this beverage helps conserve resources.	Dhir et al., 2021; Hu et al., 2022
Emotional value	Choosing this beverage makes me feel like making contribution to society. Choosing this beverage makes me feel like making contribution to sustainable development. Choosing this beverage makes me feel like doing morally right thing. Choosing this beverage makes me feel like a better person.	Roh et al., 2022
Purchase intention	I am willing to purchase this beverage. I am willing to purchase offerings of this brand. I give priority to purchasing this beverage. I would like to recommend this beverage to my friends and relatives.	Li et al., 2021; Hu et al., 2022
